# Validation of the cingulate island sign with optimized ratios for discriminating dementia with Lewy bodies from Alzheimer’s disease using brain perfusion SPECT

**DOI:** 10.1007/s12149-017-1181-4

**Published:** 2017-05-25

**Authors:** Etsuko Imabayashi, Tsutomu Soma, Daichi Sone, Tadashi Tsukamoto, Yukio Kimura, Noriko Sato, Miho Murata, Hiroshi Matsuda

**Affiliations:** 1 Integrative Brain Imaging Center, National Center of Neurology and Psychiatry, 4-1-1 Ogawahigashi, Kodaira, Tokyo 187-8551 Japan; 2 QMS Group, Quality Assurance Department, FUJIFILM RI Pharma Co., Ltd., 14-1 Kyobashi 2-Chome, Chuo-Ku, Tokyo, 104-0031 Japan; 3 Department of Radiology, Graduate School of Medicine, University of Tokyo, 3-1 Hongo 7-Chome, Bunkyo-Ku, Tokyo, 113-8655 Japan; 4 Department of Radiology, National Center of Neurology and Psychiatry, 4-1-1 Ogawahigashi, Kodaira, Tokyo 187-8551 Japan; 5 Department of Neurology, National Center of Neurology and Psychiatry, 4-1-1 Ogawahigashi, Kodaira, Tokyo 187-8551 Japan

**Keywords:** Brain perfusion SPECT, CIS, DLB, Alzheimer’s disease

## Abstract

**Objective:**

Dementia with Lewy bodies (DLB) is often cited as the second most common dementia after Alzheimer’s disease (AD). It is clinically important to distinguish DLB from AD because specific side effects of antipsychotic drugs are limited to DLB. The relative preservation of cingulate glucose metabolism in the posterior cingulate gyri versus that in the precuni, known as the cingulate island sign (CIS), in patients with DLB compared with AD is supposed to be highly specific for diagnosing DLB. In a previous study, using brain perfusion SPECT, the largest value (0.873) for the area under the receiver operating characteristic (ROC) curve (AUC) for differentiating DLB from AD was obtained with the ratio of the posterior cingulate gyri from an early Alzheimer’s disease-specific hypoperfusion volume of interest (VOI) versus the medial occipital lobe. Two purposes of this study are as follows: one is optimization of VOI setting for calculating CIS values and the other is to evaluate their accuracy and simultaneously to retest the method described in our previous paper.

**Methods:**

We conducted a retest of this SPECT method with another cohort of 13 patients with DLB and 13 patients with AD. Furthermore, we optimized VOIs using contrast images obtained from group comparisons of DLB and normal controls; the same 18 patients with DLB and 18 normal controls examined in our previous study. We obtained DLB-specific VOIs from areas where brain perfusion was significantly decreased in DLB. As the numerators of these ratios, early Alzheimer’s disease-specific VOIs were used after subtracting DLB-specific VOIs. The DLB-specific VOIs were used as the denominator.

**Results:**

In retest, the obtained AUC was 0.858 and the accuracy, sensitivity, and specificity were 84.6, 84.6, and 84.6%, respectively. The ROC curve analysis with these optimized VOIs yielded a higher AUC of 0.882; and the accuracy, sensitivity, and specificity of these new CIS ratios were 84.6, 92.3, and 76.9%, respectively, with a threshold value of 0.281.

**Conclusion:**

Optimized CISs using brain perfusion SPECT are clinically useful for differentiating DLB from AD.

## Introduction

Dementia with Lewy bodies (DLB) is often cited as the second most common dementia after Alzheimer’s disease (AD). It is clinically important to distinguish DLB from AD because specific side effects of antipsychotic drugs are limited to DLB. The relative preservation of glucose metabolism in the posterior cingulate gyri versus that in the precuni, the cingulate island sign (CIS) [1], was reported in 1997 by Imamura et al. [2] to be higher in patients with DLB than those with AD, and is highly specific for diagnosing DLB. Lim et al. [1] reported that the sensitivities for using CIS to diagnose DLB ranged from 62 to 86% and only 43 to 50% for hypometabolism in the medial occipital lobe. Recently, Graff-Radford et al. [3] reported the pathologic association of CIS with autopsy findings. These early CIS studies were limited to ^18^F-fluorodeoxyglucose positron emission tomography (^18^F-FDG-PET) imaging. O’Brien et al. [4] showed that, even with FDG, the differentiation accuracy was 72.4%, and furthermore that the CIS was not observed in SPECT images in their study. Using brain perfusion SPECT to differentiate DLB from AD [5], we previously showed that the largest value for the area under the receiver operating characteristic (ROC) curve (AUC) was 0.873; the accuracy, sensitivity, and specificity were 85.7, 88.9, and 82.4%, respectively, obtained for the ratio of the posterior cingulate gyri within early Alzheimer’s disease-specific hypoperfusion volumes of interest (VOI) in a group comparison between patients with AD and cognitively normal subjects versus the medial occipital lobe. The AUC calculated using the medial occipital VOIs was only 0.614, while the accuracy, sensitivity, and specificity were 68.6, 55.6, and 82.4%, respectively. Because AD and DLB are known to have pathological overlaps and it is difficult to distinguish these two diseases using ^18^F-FDG-PET or brain perfusion SPECT [4], it was logical to use these early AD-specific VOIs to discriminate AD from DLB [5].

Therefore, in the current study to optimize these VOIs and to verify the clinical usefulness of CIS in different groups of patients [i.e., group A with DLB from our previous study, group B with DLB from the present study and group C with AD from the present study (Table 1)], we first compared a group of patients with DLB (group A in Table 1) to normal controls to obtain specific VOIs. We then applied these optimized VOIs to new groups (group B and C in Table 1) of patients with DLB and AD to evaluate their accuracy using ROC curve analysis and to determine the thresholding CIS values to discriminate DLB from AD.

**Table 1:** Demographic data

Characteristic	Normal controls	DLB		AD
		Group A	Group B	Group C
Age at the time of SPECT	73.9 ± 6.9	73.9 ± 6.8	76.3 ± 6.4	72.5 ± 8.6
Male/female	10/8	10/8	6/7	3/10
MMSE	*	19.1 ± 6.7	21.7 ± 6.6	21.5 ± 3.3
Heart to mediastinum ratio in [^123^I]MIBG (3 h after injection)	NA	1.33 ± 0.14 (1.09–1.62)	1.42 ± 0.19 (1.15–1.80)	NA

* every normal subject's MMSE score was 26 or more

*AD* Alzheimer’s disease, *DLB* dementia with Lewy bodies, *SPECT* single-photon emission computed tomography, *MMSE* Mini Mental State Examination, *[* ^*123*^ *I]MIBG* [^123^I]metaiodobenzylguanidine

Accordingly, two purposes of this study are as follows: one is optimization of VOI setting for calculating CIS values by setting statistically significant hypoperfusion areas as VOIs for both denominators and numerators and the other is to evaluate their accuracy and simultaneously to retest the method described in our previous paper [5].

## Materials and methods

### Subjects

This single-center, retrospective study was conducted in accordance with the tenets of the Declaration of Helsinki, and the use of previously obtained images with public notification was approved by the ethical committee of our institute. Thirty-one patients with DLB (M:F 16:15, age 74.9 ± 6.7 years), 13 ^11^C-PiB-positive patients with AD (M:F 3:10, age 72.5 ± 8.6 years), and 18 cognitively normal subjects (M:F 10:8, age 73.9 ± 6.9 years) were studied. The 31 patients with DLB consisted of two groups: 18 subjects with DLB (group A) (M:F 10:8, age 73.9 ± 6.8 years) were the same patients described in our previous paper [5], and 13 other patients (group B) (M:F 7:6, age 76.6 ± 6.5 years). The 18 normal subjects were semi-randomly selected to be age- and gender-matched to the subjects with DLB in group A from a previous study performed in our institute [6]. The patients with DLB in group B were chosen from those who underwent [^123^I] metaiodobenzylguanidine (MIBG) myocardial scintigraphy and who fulfilled the criteria of probable DLB proposed in the third consortium on DLB international workshop [7] from chart screenings. The 13 AD subjects were selected from another of our studies after providing informed consent [8]. These previous studies were approved by the Ethics Committee for Clinical Research in our institute and informed, written consent was obtained from all subjects.

### Brain perfusion SPECT

All subjects were asked to remain in a comfortable, supine position with their eyes closed in dark, quiet surroundings. An intravenous injection of 740 MBq [^99m^Tc] ethyl cysteinate dimer (ECD; Fujifilm RI Pharma, Tokyo, Japan) was administered. Ten minutes later, a SPECT scan was obtained using a 2-head gamma camera and 6-slice CT system (Symbia T6; Siemens, Erlangen, Germany) equipped with low-energy, high-resolution, and parallel-hole collimators. Ninety views were obtained continuously throughout 360° of rotation (4°/step, 128 × 128 matrix, zoom 1.45). The voxel size was 3.3 × 3.3 × 3.3 mm. To reconstruct the SPECT image, a combination of Fourier rebinning followed by ordered subset expectation–maximization (iteration number 8 and subset 10) and a 7-mm full width at half maximum Gaussian filter were used. To reconstruct the image by fitting it to a normal data base, Chang’s method [9] was used for attenuation correction.

### Group comparisons

**Fig. 1:**
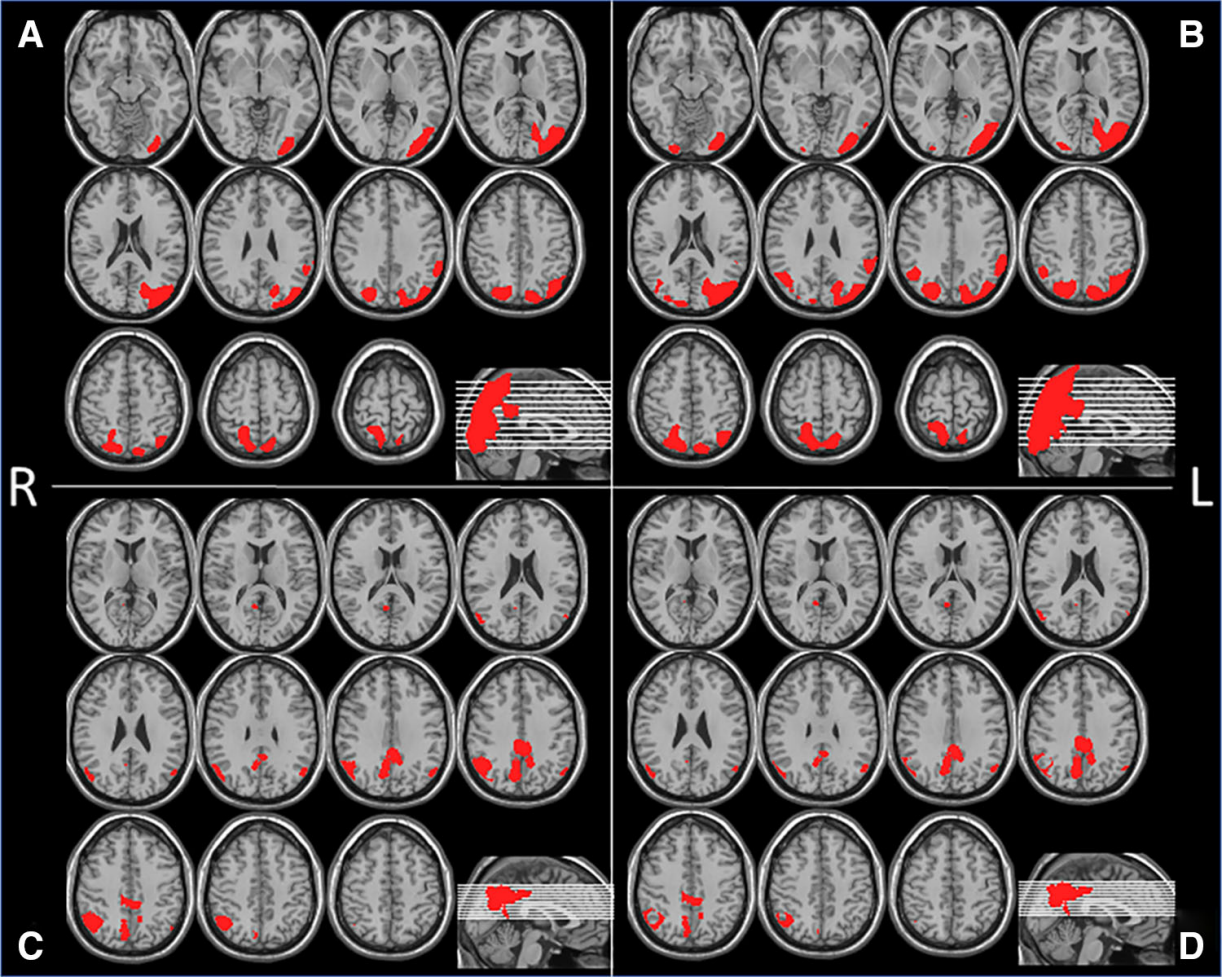
**a**, **b** Group analysis of gray matter accumulation between the DLB and normal controls was performed using a spatial extent threshold of 1000 for contiguous voxels. Main effects used whole-brain analyses with a threshold at a voxel level of family-wise error (FWE) of *p* < 0.01 or *p* < 0.05. These contrast maps DLB_specific_VOI_1 (**a**) for *p* < 0.01, DLB_specific_VOI_2 (**b**) for *p* < 0.05. **c** AD_DLB_num_1, which is equal to AD-specific VOI in eZIS software minus DLB_specific_VOI_1, **d** AD_DLB_num_2, which is equal to AD-specific VOI in eZIS software minus DLB_specific_VOI_2

To demarcate the areas where specific hypoperfusion is observed in DLB patients, a group comparison between the 18 DLB subjects in group A and the 18 normal subjects was conducted using statistical parametric mapping (SPM) 12 (http://www.fil.ion.ucl.ac.uk/spm/), which implements the general linear model; statistically significant hypoperfusion areas were then extracted from this comparison. Proportional scaling was used to achieve global normalization of voxel values among the images. We studied the differences in gray matter perfusion between these two groups using *t* statistics. The resulting sets of *t* values constituted statistical parametric maps: SPM (*t*) that were transformed to the unit normal distribution (SPM[*Z*]). Group analysis of gray matter accumulation between the DLB and normal controls was performed using a spatial extent threshold of 1000 for contiguous voxels. Main effects used whole-brain analyses with a threshold at a voxel level of family-wise error correction (FWE) of *p* < 0.01 or *p* < 0.05. These contrast maps were saved as “DLB_specific_VOI_1” for *p* < 0.01 (Fig. 1a) and “DLB_specific_VOI_2” for *p* < 0.05 (Fig. 1b) and “tDLB_specific_VOI_1” and “tDLB_specific_VOI_2” were defined as total positive *Z* score values within these VOIs, respectively.

### Image preprocessing

*Z* score maps of the obtained SPECT images of the 13 patients with DLB in group B and 13 normal controls were converted using the easy *Z* score imaging system (eZIS) analysis (Fujifilm RI Pharma Co., Ltd., Tokyo, Japan) software [10]. It included spatial normalization parameters in SPM2 and a [^99m^Tc] ECD brain template in the same space as the Montreal Neurological Institute (MNI) standard brain template [11]. Normal databases were included in the eZIS and inter-institutional differences were corrected using previously scanned phantom data [12].

After the inter-institutional correction, specially normalized [^99m^Tc]ECD SPECT images from each patient were compared with normal images from the database; ECD60-69yDB (the database of 60–69-year-olds), ECD70y-DB (the database of over 70-year-olds), using voxel-by-voxel *Z* score analysis after pixel normalization to the global mean values [*Z* score = [(control mean) – (individual value)]/(control SD)] as previously reported by Minoshima et al. [13].

### Volumes of interest

The eZIS software included a set of AD-specific hypoperfusion VOIs identified in patients with AD following a group comparison with cognitively healthy individuals [14]. The contrast maps obtained from the group comparison described above (DLB_specific_VOI_1 and DLB_specific_VOI_2) were then subtracted from the AD-specific hypoperfusion areas originally included in the eZIS software (Fig. 1c, d). We named these VOIs “AD_DLB_num_1” and “AD_DLB_num_2” and these CIS ratio numerator “tAD_DLB_num_1” and “tAD_DLB_num_2” were defined as total positive *Z* score values within these VOIs, respectively, that is, the AD_DLB_num_1 is equal to the AD-specific VOI minus the DLB_specific_VOI_1, and AD_DLB_num_2 is equal to the AD-specific VOI minus DLB_specific_VOI_2. We also investigated VOIs described in our previous paper as “PCG_AD_VOIs” and “tPCG_AD_VOIs” was defined as total positive *Z* score values within these VOIs, which were overlap areas of significant perfusion reduction identified in patients with AD following a group comparison with cognitively healthy individuals [14] and also included in the eZIS software and posterior cingulate gyrus in automated anatomical labeling (AAL). Furthermore, we investigated the posterior cingulate VOI included in the AAL atlas. This was named “AAL_PCG” and” tAAL_PCG” was defined as total positive *Z* score values within these VOIs. To determine the CIS ratios of the above, as denominator we used DLB_specific_VOI_1 and DLB_specific_VOI_2, medial occipital VOI in AAL (AAL_medOccipital), and precuneus and cuneus VOIs in AAL (AAL_PreC&C) and “tAAL_medOccipital” and “tAAL_PreC&C” were defined as total positive *Z* score values within these VOIs, respectively. This AAL_PreC&C VOI was used to calculate the original CIS for the FDG-PET analysis [1, 3]. We then calculated a total positive *Z* score in each VOI and divided the numerator by the denominator.

### Receiver operating characteristic (ROC) curve analysis

The AUC of the ROC curve was obtained by thresholding with each of these values for all VOIs. Finally, the AUCs were statistically compared [15] (Table 2). The AUC value of ≥0.87 demonstrated moderate discriminatory power [16] and, therefore, optimal thresholds were determined that supplied the maximum number of true positive and true negative subjects. Accuracy, sensitivity, and specificity resulting from that threshold were then calculated (Table 3).

**Table 2:** Area under the receiver operating characteristic (ROC) curve (AUC) for various combinations of ratio numerators and denominators

VOIs	Numerator			
	AD_DLB_num_1	AD_DLB_num_2	PCG_AD_VOI	AAL_PCG
Denominator				
DLB_specific_VOI_1	**0.876** **±** **0.0681** (0.687–0.971)	**0.882** **±** **0.0682** (0.695–0.974)	0.757 ± 0.0973 (0.551–0.902)	0.840 ± 0.0803 (0.644–0.953)
DLB_specific_VOI_2	0.864 ± 0.0725 (0.673–0.966)	0.852 ± 0.0793 (0.658–0.960)	0.740 ± 0.100 (0.532–0.890)	0.817 ± 0.0867 (0.17–0.960)
AAL_medOccipital	0.864 ± 0.0785 (0.673–0.966)	0.858 ± 0.0797 (0.666–0.963)	0.858 ± 0.0773 (0.666–0.963)	**0.882** **±** **0.0730** (0.695–0.974)
AAL_PreC&C	**0.899** **±** **0.0608** (0.717–0.982)	**0. 888** **±** **0.0635** (0.702–0.977)	0.763 ± 0.0977 (0.557–0.906)	0.793 ± 0.0908 (0.590–0.925)

Values in bold indicate AUCs ≥0.87. AUC ± standard error (95% confidential interval)

*AD* Alzheimer’s disease, *DLB* dementia with Lewy bodies, *VOI* volume of interest, *AAL* automated anatomical labeling, *PCG* posterior cingulate gyrus, *PreC&C* precuneus and cuneus

**Table 3:** Accuracy, sensitivity, and specificity of area under the receiver operating characteristic (ROC) curve (AUC) demonstrating moderate discrimination of DLB from AD

Numerator	Denominator	AUC	Accuracy (%)	Sensitivity (%)	Specificity (%)	Threshold
tAD_DLB_num_1	tDLB_specific_VOI_1	0.876	80.8	69.2	92.3	0.259
tAD_DLB_num_2^a^	tDLB_specific_VOI_1^a^	0.882	**84.6**	**92.3**	**76.9**	**0.281**
tAD_DLB_num_1^b^	tAAL_PreC&C^b^	0.899	**84.6**	**84.6**	**84.6**	**0.348**
tAD_DLB_num_2	tAAL_PreC&C	0.888	80.8	69.2	92.3	0.221
tAAL_PCG^c^	tAAL_medOccipital^c^	0.882	**84.6**	**76.9**	**92.3**	**0.0308**

Values in bold indicate the highest accuracy and associated parameters

*AD* Alzheimer’s disease, *DLB* dementia with Lewy bodies, *VOI* volume of interest, *AAL* automated anatomical labeling, *PCG* posterior cingulate gyrus, *PreC&C* precuneus and cuneus

^a^tAD_DLB_num_2/tDLB_specific_VOI_1; CISRo; CIS ratio optimized

^b^tAD_DLB_num_1/tAAL_PreC&C: CISRmix; CIS ratio optimized and with AAL

^c^tAAL_PCG/tAAL_medOccipital; CISRaal; CIS ratio with AAL

## Results

The patient characteristics are summarized in Table 1. No significant differences were observed for age or gender. All the heart to mediastinum ratios in the [^123^I] MIBGs scanned 3 h after injection in patients with DLB were below 2.0.

**Fig. 2:**
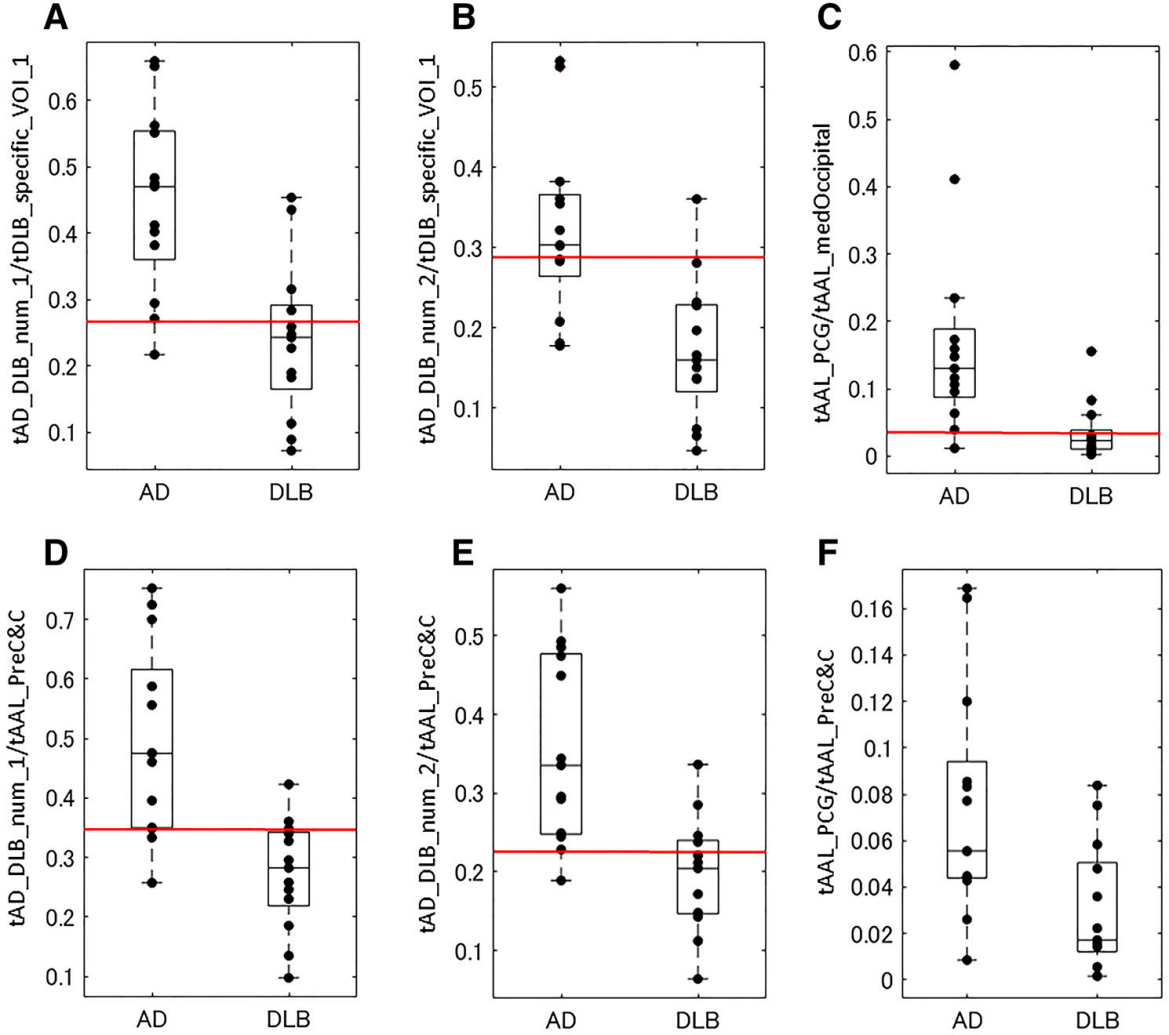
Box plots of CIS ratios with optimized VOIs and AAL VOIs. **a** tAD_DLB_num_1/tDLB_specific_VOI_1; **b** CISRo: tAD_DLB_num_2/tDLB_specific_VOI_1; **c** CISRaal: tAAL_PCG/tAAL_medOccipital; **d** CISRmix: tAD_DLB_num_1/tAAL_PreC&C; **e** tAD_DLB_num_2/tAAL_PreC&C; **f** CIS: tAAL_PCG/tAAL_PreC&C. The *boxes* indicate the upper and lower quartiles with the median, and the *whiskers* show the minimum, maximum, or 1.5 × interquartile ranges. The *red lines* on **a**–**e** indicate the thresholds shown in Table 3

As shown in Table 2, there were no significant differences among any of the analysed AUCs. Combinations of these that resulted in AUCs ≥0.87 are listed in Table 3. A higher accuracy of 84.6% was obtained with three VOI combinations for numerator and denominator when the ratio sum of all the positive *Z* scores within those VOIs was used as thresholds. The first of these utilized the optimized VOIs: tAD_DLB_num_2/tDLB_specific_VOI_1, which was termed as “CISRo” (CIS ratio optimized); the second the mixed VOIs: tAD_DLB_num_1/tAAL_PreC&C, which was termed as “CISRmix” (CIS ratio optimized and with AAL); and the third the AAL VOIs: tAAL_PCG/tAAL_medOccipital, which was termed as “CISRaal” (CIS ratio with AAL). The accuracy, sensitivity, and specificity of the CISRo for differentiating patients with DLB from those with AD were 84.6, 92.3, and 76.9%, respectively, with a threshold value less than 0.281; for the CISmix, they were 84.6, 84.6, and 84.6%, respectively, with a threshold value less than 0.348; and for the CISRaal, they were 84.6, 76.9, and 92.3%, respectively, with a threshold value less than 0.0308. Box plots of these CIS ratios with optimized VOIs and AAL VOIs were in Fig. 2.

## Discussion

The CIS, the relative preservation demonstrated in ^18^F-FDG-PET of glucose metabolism in the posterior cingulate gyri versus that in the precuni, in patients with DLB compared with those with AD is considered to be highly specific for diagnosing DLB [1]. In our previous study [5] of brain perfusion SPECT using [^99m^Tc] ECD, we also observed a differential CIS ratio when the method was optimized using the posterior cingulate area of an early AD-specific VOI included in eZIS software as numerator and the anatomical medial occipital area as denominator for this ratio. Because brain perfusion SPECT is relatively more economical and widely available worldwide than PET, the clinical application of CIS to differentiate patients with DLB from AD would be more effective using brain perfusion SPECT. In the current study, not only did we determine the threshold value for clinical use, but we also tried to optimize the CIS in brain perfusion SPECT and at the same time to retest it in another patient cohort. As a result, we obtained a larger AUC than that of our previous study [5].

Previously, we used the posterior cingulate area within the early AD-specific VOI as numerator, and did not take into account a DLB-specific VOI. When we used the same numerator in the current retest study that previously produced the largest AUC, we received an AUC of 0.858. Although this was smaller than the previous AUC value of 0.873, it still fell within the 95% confidence interval. Also the accuracy (84.6 versus 85.7%) and sensitivity (84.6 versus 88.9%) of the retest were slightly lower than those of the previous study; however, also all of these values are over 84% and thus appear to be reliable enough to evaluate the differentiation of DLB from AD.

In the original CIS study [1], AAL_PCG VOI was used as a numerator and AAL_PreC&C VOI as a denominator, resulting in accuracy, sensitivity, and specificity of 78, 77, and 80%, respectively. These ratios were also used as thresholds, and ROC analysis was applied in the current study, which resulted in accuracy, sensitivity, and specificity of 76.9, 61.5, and 92.3%, respectively. These results indicate that even though the resolution of SPECT is generally inferior to those of PET, the accuracy, sensitivity, and specificity obtained in the current study were comparable to those obtained with PET. Furthermore, we also effectively optimized the VOI parameters.

DLB is often cited as the second most common dementia after AD. It is clinically important to distinguish DLB from AD because specific side effects of antipsychotic drugs are limited to DLB. In discriminating DLB from AD clinically, dopamine transporter (DAT) imaging [17] and [^123^I] MIBG [18] are useful because they detect early disturbances of the nigrostriatal pathway or peripheral sympathetic nervous system in patients with DLB. With a combination of these two techniques, over 90% sensitivity and specificity in discriminating DLB from AD are reported [19]. Nevertheless, brain perfusion SPECT is more widely and commonly used for clinical screening of patients with dementia. Furthermore, compared with morphometric imaging, SPECT is a more sensitive modality for functional imaging used to detect early stages of neurodegenerative disease before shrinkage [20]. SPECT also reveals useful information for differentiating AD as well as other dementias, including vascular dementia or frontotemporal lobe degeneration [21].

In this study, in trying to optimize the use of the CIS in SPECT, we subtracted DLB-specific VOIs derived from comparisons between DLB and normal controls from AD-specific VOIs. Furthermore, the CIS ratio was determined by comparing these with specific hypoperfusion areas in DLB instead of the entire anatomical occipital area. This resulted in a higher AUC of 0.882 compared with our previous study [21]. With these optimized numerators and denominators determining the CIS ratio, we obtained the largest specificity of 92.3% for differentiating DLB from AD. Hypothetically, if we used this procedure with highly specific examinations such as [^123^I] MIBG [22] or DAT imaging [19], an even higher accuracy might be achieved. Moreover, a combination of these two with brain perfusion SPECT might prove to be even more valuable, considering the purpose of the screening, compared with using only MIBG and DAT, which only detect degeneration of central or peripheral monoamine pathways, while brain perfusion SPECT measures whole brain activity and can differentiate many brain disorders.

Contrary to practical clinical situations, amyloid PET was used as a biomarker for diagnosing AD in this study. As a subject of important future investigation, additional clinical prospective study using our CIS procedure for diagnosing AD without amyloid PET may reinforce the effective diagnostic flowchart with combination of the CIS, amyloid PET, MIBG and DAT imaging.

The CIS observed in ^18^F-FDG-PET was reportedly indicative of a lower Braak neurofibrillary tangle stage in patients with DLB [3]. In our study, we compared the CIS detected using brain perfusion SPECT in patients with DLB and AD. While we cannot compare these two studies directly, brain perfusion and metabolism are physiologically coupled [23]. The limitations of SPECT include lower image resolution and a large partial volume effect. These limitations, in conjunction with a patient’s pathological status, result in decreased accuracy when attempting to differentiate between DLB and AD. However, optimization of the procedure using the CIS ratio described in this paper seems to overcome these limitations and achieve a larger accuracy compared with the existing procedure. Furthermore, SPECT is widely accessible and more economical than PET.

## Conclusion

To further the clinical usage of CIS to discriminate between DLB from AD, we retested and optimized the procedure, and thereby achieved a larger AUC of 0.882. In so doing, the accuracy, sensitivity, and specificity were 84.6, 92.3, and 76.9%, respectively, with a threshold value less than 0.281 for CISRo, 84.6, 84.6, and 84.6%, respectively, with a threshold value less than 0.348 for CISRmix, and 84.6, 76.9, and 92.3%, respectively, with a threshold value less than 0.03 for CISRaal. Brain perfusion SPECT is not only useful in screening for dementia and economically and widely accessible, but also can facilitate the differentiation of DLB from AD when these new CIS ratios are properly used.
